# Endoscopic Laryngeal Findings in Japanese Patients with Laryngopharyngeal Reflux Symptoms

**DOI:** 10.1155/2012/908154

**Published:** 2011-11-20

**Authors:** Nobuhiko Oridate, Ryoji Tokashiki, Yusuke Watanabe, Aki Taguchi, Osamu Kawamura, Kazuma Fujimoto

**Affiliations:** ^1^Department of Otolaryngology-Head and Neck Surgery, Hokkaido University Graduate School of Medicine, Kita 15, Nishi 7, Kita-ku, Sapporo 060-8638, Japan; ^2^Department of Otolaryngology, Tokyo Medical University, Tokyo 160-0023, Japan; ^3^Department of Otorhinolaryngology, International University of Health and Welfare Mita Hospital, Tokyo 108-8329, Japan; ^4^Department of Otolaryngology, Ehime University Graduate School of Medicine, Ehime 791-0295, Japan; ^5^Department of Gastroenterology, Gunma University Hospital, Maebashi, Gunma 371-8511, Japan; ^6^Department of Internal Medicine, Saga Medical School, Saga 849-8501, Japan

## Abstract

*Objective*. To know the characteristics of endoscopic laryngeal and pharyngeal abnormalities in Japanese patients with laryngopharyngeal reflux symptoms (LPRS). *Methods*. A total of 146 endoscopic images of the larynx and pharynx (60 pairs for the rabeprazole group and 13 pairs for the control group) were presented to 15 otolaryngologists blinded to patient information and were scored according to several variables potentially associated with laryngopharyngeal reflux. The median value of the 15 scores for each item from each image was obtained. The mean pretreatment scores of each item and total score were assessed in both rabeprazole and control groups. In the rabeprazole group, the endoscopic findings before and after the 4-week treatment with rabeprazole were compared. Changes between corresponding duration in the control group were also evaluated. *Results*. The median and mean pretreatment total score was 3 and 3.02, respectively, from the 73 patients with LPRS. No significant differences were observed before and after treatment in either the rabeprazole or control groups for any item or total score. In 24 patients with a high pretreatment score (total score ≥ 4) from the rabeprazole group, significant decreases in scores for “thick endolaryngeal mucous” (0.54 to 0.17, *P* = 0.017) and total (4.77 to 3.58, *P* = 0.0003) were observed after the 4-week treatment.

## 1. Introduction

Gastroesophageal reflux is a recognized cause of ENT symptoms [1]. Laryngopharyngeal symptoms and signs were referred to as laryngopharyngeal reflux (LPR) [2]. The laryngopharyngeal findings attributed to gastroesophageal reflux have been reported in the posterior pharyngeal wall, true vocal folds, and arytenoid medial wall [3]. However, accurate assessment of signs in the larynx and pharynx is likely to be difficult because these signs observed during a laryngoscopic examination cannot be reliably determined from clinician to clinician [4]. The sensitivity and specificity of laryngopharyngeal findings, therefore, remain uncertain, challenging the diagnostic accuracy of LPR. In this study, we conducted a multicenter clinical trial to explore the presence of endoscopic laryngeal findings in Japanese patients with laryngopharyngeal reflux symptoms (LPRSs). Because the first-line therapy for LPRS is considered to be proton pump inhibitor (PPI) [2], we also compared the endoscopic laryngeal findings before and after a 4-week acid suppression therapy.

## 2. Methods

Subjects consisted of outpatients visiting the otolaryngology departments of participating institutions between October 2007 and May 2008 who had at least one LPRS such as lump in the throat, throat pain, irritation in the throat, chronic cough, and hoarseness and whose consent could be obtained. A total of 255 endoscopic laryngeal images were presented to 15 otolaryngologists listed in the appendix with the subjects' names and their before and after therapy status blinded. The 15 otolaryngologists individually scored on a four-point scale as 0 (none), 1 (mild), 2 (moderate), and 3 (severe) or NE (not evaluable) for findings potentially associated with LPR, as shown in Table 1. First 5 of 7 items are derived from the Reflux Finding Score proposed by Belafsky et al. [5] and the other 2 were from the report by Vaezi et al. [3].

**Figure 1:**
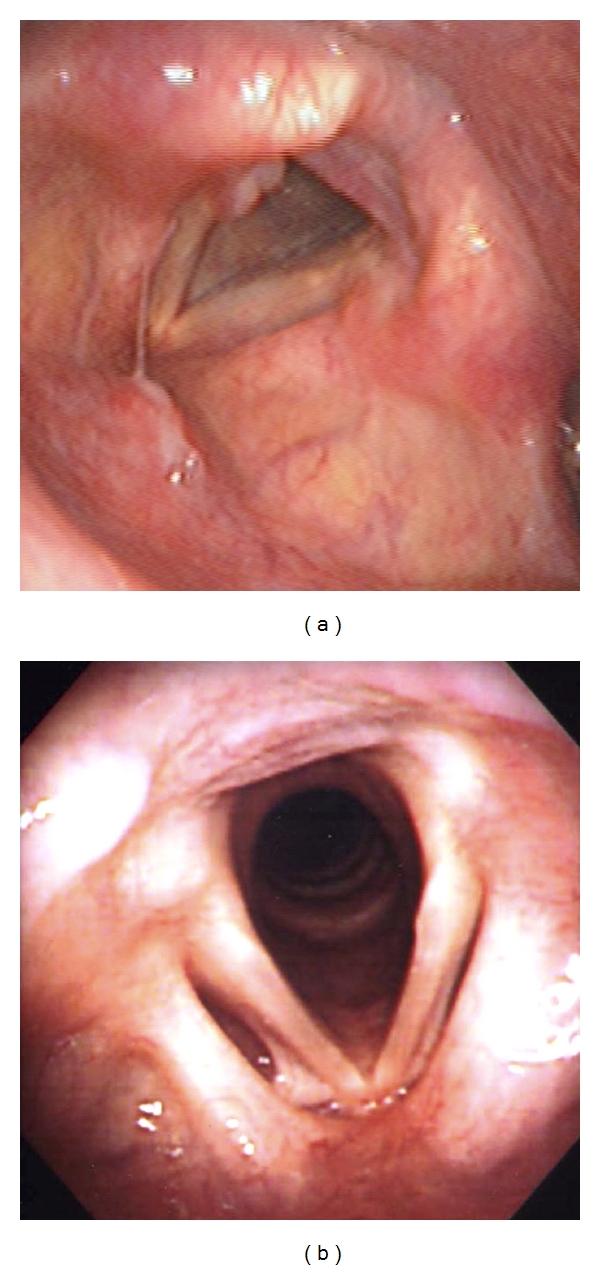
Examples for endoscopic laryngeal images with (a) a high score (total score: 7) and (b) a lowest score (total score: 0).

Of the 255 images, 109 were excluded (95, patient overlap; 14, number of NE items > 3), and the remaining 146 images were used for further analysis. The median value of the 15 scores for each item from each subject was obtained. The mean pretreatment scores of each item and total scores were assessed. Examples for the images with high (total score: 7) and low (total score: 0) median scores were shown in Figures 1(a) and 1(b), respectively. To 60 patients who were considered for indication of acid suppression therapy based on their symptoms, 10 mg/day of rabeprazole (RPZ) for 4 weeks was administered and the endoscopic findings before and after the 4-week treatment with RPZ were compared. Changes between corresponding duration in 13 patients, who had at least one LPRS and had not received acid suppression therapy, were also evaluated. Double-sided paired or unpaired *t*-tests were used to test the significance of differences.

## 3. Results

The pretreatment total score for all 73 subjects ranged from 0 to 7 (median score 3, mean score 3.02). No significant differences were observed between the groups for any item or total score (RPZ group: 3.12; control group: 2.54, Table 2). Further, no significant differences were observed before and after treatment in either the RPZ or control groups for any item or total score (Tables 3 and 4). In 24 patients with a high pretreatment score (total score ≥ 4) from the RPZ group, significant decreases in scores for “thick endolaryngeal mucous” (0.54 to 0.17, *P* = 0.017) and total (4.77 to 3.58, *P* = 0.0003) were observed after the 4-week treatment (Table 5).

## 4. Discussion

The precise laryngoscopic diagnosis of LPR is likely to be difficult because the examination of abnormalities in the larynx and the pharynx could be highly subjective [4]. Even with using gastrointestinal endoscopy which provides clear images with higher resolution than laryngoscopy, diagnostic value was limited when evaluating these laryngopharyngeal lesions in patients with gastroesophageal reflux [6]. Some authors emphasize these findings in the larynx and pharynx as being specific for acid-related problems, others argue that these may be secondary to other factors such as smoking, allergies, asthma, viral illness, and voice abuse [3, 7, 8].

During our daily practice in the ENT clinics, we noticed that most of Japanese patients who complain a typical constellation of LPRS do not necessarily exhibit such laryngopharyngeal abnormal findings. We also noticed that minimal changes of these findings are very difficult to be documented objectively. These small abnormalities cannot be revealed due to the consideration to examiner bias [4, 6]. We performed this study to ensure objectivity to some extent of the examination of abnormalities in the larynx and pharynx in this patient population. We presumed that the median value of 15 otolaryngologists was the most appropriate value of each finding of the laryngopharyngeal abnormality. We then found a low pretreatment score among the Japanese patients with LPRS, suggesting that most of them had only mild laryngeal signs. When limited to the patients with a high endoscopic laryngeal score, a significant decrease in total score was observed after acid-suppression therapy.

There are some limitations in this study. Among them, the major one would be the length and the dose of PPI treatment. These could be a possible reason for causing no significant difference before and after the RPZ treatment. The 4 weeks of acid suppression with RPZ with a dose of 10 mg/day may not be long or strong enough to see objective improvement. Ford proposed an empirical therapeutic trial using double-dose, twice-daily PPI for three months [2], suggesting that both the length and the dose of PPI treatment in this study were not enough to observe significant changes in the laryngopharyngeal findings.

Currently, only the patients with obvious laryngopharyngeal abnormalities were recruited to the prospective, randomized, double-blind and placebo-controlled studies on the effect of PPI on symptom improvement [9, 10]. In Lam's report, there were no significant differences in laryngopharyngeal findings between the PPI and placebo groups, suggesting that the improvement in laryngeal signs might not lead to significant improvement in patient symptoms. In other words, laryngeal signs may not correlate faithfully with actual improvement in LPRS. It may be possible to postulate that the effect of PPI is not limited to the patients with obvious laryngopharyngeal abnormalities. The correlation between laryngopharyngeal symptoms and signs would need further studies. Because the precise diagnosis of LPR is still difficult, it is of critical importance to identify morphologic or physiologic features more specific for LPR.

## 5. Conclusions

The low pretreatment total score of the Japanese patients with LPRS suggested that most of them had only mild objective laryngeal signs. In LPRS patients with a high endoscopic laryngeal score, a significant decrease in total score was observed after acid suppression therapy.

## Figures and Tables

**Table 1:** Score for endoscopic laryngeal findings used in this study.

Findings	Score				
Infraglottic edema with pseudosulcus formation	0	1	2	3	NE
Laryngeal mucosa ledema	0	1	2	3	NE
Posterior commissure hypertrophy	0	1	2	3	NE
Granulation formulation	0	1	2	3	NE
Thick endolaryngeal mucous	0	1	2	3	NE
Redness in the intra-arythenoid medial wall	0	1	2	3	NE
Mucous pooling in the pyriform sinus	0	1	2	3	NE

NE: not evaluable.

**Table 2:** Mean scores for pretreatment endoscopic findings (*n* = 73).

Endoscopic findings	Total (*n* = 73)	RPZ (*n* = 60)	Control (*n* = 13)	*P*-value
Infraglottic edema with pseudosulcus formation	0.39	0.40	0.35	0.776

Laryngeal mucosa edema	0.52	0.50	0.62	0.471

Posterior commissure hypertrophy	0.83	0.88	0.62	0.106

Granulation formulation	0.15	0.17	0.08	0.362

Thick endolaryngeal mucous	0.22	0.23	0.15	0.523

Redness in the intra-arythenoid medial wall	0.49	0.53	0.31	0.153

Mucous pooling in the pyriform sinus	0.43	0.43	0.42	0.968

Total	3.02	3.13	2.54	0.258

**Table 3:** Endoscopic findings before and after 4 weeks in the control group (*n* = 13).

Endoscopic findings	Initial	4 weeks later	*P*-value
Infraglottic edema with pseudosulcus formation	0.35	0.46	0.570

Laryngeal mucosa edema	0.62	0.77	0.337

Posterior commissure hypertrophy	0.62	0.62	1.000

Granulation formulation	0.08	0.08	1.000

Thick endolaryngeal mucous	0.15	0.31	0.337

Redness in the intra-arythenoid medial wall	0.31	0.15	0.337

Mucous pooling in the pyriform sinus	0.42	0.50	0.838

Total	2.54	3.00	0.239

**Table 4:** Pre- and posttreatment endoscopic findings in the RPZ group (*n* = 60).

Endoscopic findings	Pretreatment	Post-treatment	*P*-value
Infraglottic edema with pseudosulcus formation	0.40	0.33	0.419

Laryngeal mucosa edema	0.50	0.59	0.268

Posterior commissure hypertrophy	0.88	0.98	0.147

Granulation formulation	0.17	0.18	0.709

Thick endolaryngeal mucous	0.23	0.15	0.279

Redness in the intra-arythenoid medial wall	0.53	0.62	0.273

Mucous pooling in the pyriform sinus	0.43	0.37	0.279

Total	3.13	3.20	0.779

**Table 5:** Pre- and post-treatment endoscopic findings in patients with a total score ≥ 4 from the RPZ group (*n* = 24).

Endoscopic findings	Pretreatment	Post-treatment	*P*-value
Infraglottic edema with pseudosulcus formation	0.58	0.38	0.203

Laryngeal mucosa edema	0.79	0.65	0.307

Posterior commissure hypertrophy	1.10	0.96	0.166

Granulation formulation	0.29	0.29	1.000

Thick endolaryngeal mucous	0.54	0.17	0.017

Redness in the intra-arythenoid medial wall	0.83	0.73	0.396

Mucous pooling in the pyriform sinus	0.63	0.42	0.135

Total	4.77	3.58	0.0003
